# A Randomized Pharmacokinetic Study of Generic Tacrolimus Versus Reference Tacrolimus in Kidney Transplant Recipients

**DOI:** 10.1111/j.1600-6143.2012.04174.x

**Published:** 2012-10

**Authors:** R R Alloway, B Sadaka, J Trofe-Clark, A Wiland, R D Bloom

**Affiliations:** a Division of Nephrology, Section of Transplantation, University of CincinnatiCincinnati, OH; b Renal Division, Perelman School of Medicine, University of Pennsylvania, and Department of Pharmacy, Hospital University of PennsylvaniaPhiladelphia, PA; c Novartis Pharmaceuticals CorporationEast Hanover, NJ; d Renal Division, Perelman School of Medicine, University of PennsylvaniaPhiladelphia, PA

**Keywords:** Generic, Hecoria, kidney transplantation, pharmacokinetic, Sandoz, tacrolimus

## Abstract

Pharmacokinetic analyses comparing generic tacrolimus preparations versus the reference drug in kidney transplant patients are lacking. A prospective, multicenter, open-label, randomized, two-period (14 days per period), two-sequence, crossover and steady-state pharmacokinetic study was undertaken to compare twice-daily generic tacrolimus (Sandoz) versus reference tacrolimus (Prograf®) in stable renal transplant patients. AUC_0–12h_ and peak concentration (C_max_) were calculated from 12 h pharmacokinetic profiles at the end of each period (days 14 and 28). Of 71 patients enrolled, 68 provided evaluable pharmacokinetic data. The ratios of geometric means were 1.02 (90% CI 97–108%, p = 0.486) for AUC_0–12h_ and 1.09 (90% CI 101–118%, p = 0.057) for C_max_. Mean (SD) C_0_ was 7.3(1.8) ng/mL for generic tacrolimus versus 7.0(2.1) ng/mL for reference tacrolimus based on data from days 14 and 28. Correlations between 12 h trough levels and AUC were r = 0.917 for generic tacrolimus and r = 0.887 for reference drug at day 28. These data indicate that generic tacrolimus (Sandoz) has a similar pharmacokinetic profile to the reference drug and is bioequivalent in kidney transplant recipients according to US Food and Drug Administration and European Medicines Agency guidelines.

## Introduction

Since expiry of the tacrolimus patent in 2008, generic preparations have become available and have been widely adopted. However, tacrolimus pharmacokinetics are relatively complex with a high degree of inter- and intrapatient variability such that therapeutic drug monitoring is mandatory. Differences between patients (interpatient variability) can be affected by a multitude of factors, including patient demographics, liver function, diurnal variation, concomitant immunosuppressants, gastrointestinal disturbances, coexisting diabetes mellitus and genetic differences in CYP3A4 and P-glycoprotein expression (1). In transplant patients, the key contributors to intrapatient variability in immunosuppressant dosing are usually drug–drug, drug–disease and food–drug interactions. Against this background, careful examination of generic tacrolimus preparations compared to the reference preparation (Prograf®) is essential to ensure that exposure is similar on substitution in stable renal transplant patients.

Regulatory approval of generic products requires only evidence of equivalent relative oral bioavailability versus the originator drug in healthy volunteers, studies that are generally performed in small populations using a single-dose, two-way crossover design (2). Renal transplant patients, however, exhibit a higher rate of tacrolimus clearance than healthy volunteers (3), possibly due to low hematocrit and albumin levels, concomitant administration of corticosteroids (4) and high rates of disturbed gastrointestinal motility and diabetes. Thus, robust pharmacokinetic data in the kidney transplant population would be highly relevant to physicians considering adoption of a generic formulation (5,6), and both the American (7) and European (8) transplant societies as well as other expert groups (9, 10) have noted the limitations of extrapolating data from healthy volunteers to transplant populations. Area under the curve (AUC) concentration measurements act as a marker for the extent of absorption, while peak concentration (C_max_) and time to C_max_ (t_max_) characterize the rate of absorption. The Food and Drug Administration (FDA) requires that the 90% confidence interval (CI) of the ratio of the geometric means for the generic compared with the originator falls between 80% and 125% for both AUC and C_max_ (11). The European Medicines Agency (EMA) stipulates a slightly narrower window for AUC (90–111%) (12). Under the EMA guidelines, only AUC_0-t_ is always required, while C_max_ assessment is only necessary when relevant (i.e. if it is of particular importance for safety, efficacy or drug-level monitoring). To date, bioequivalence testing of generic tacrolimus preparations has not been undertaken in kidney transplant recipients.

We report here the findings of a prospective, multicenter, open-label, randomized crossover study undertaken with the objective of comparing the steady-state pharmacokinetics of a generic tacrolimus formulation (Sandoz) versus the originator drug (Prograf®) in stable renal transplant patients.

## Methods

### Study design

**Figure 1:**
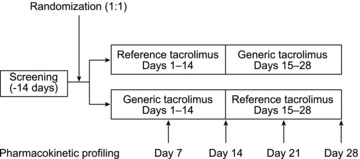
Study design

This was an open-label, prospective, multicenter, randomized, two-period, two-sequence crossover, steady-state pharmacokinetic study conducted during October 2010–May 2011 (Figure 1). During a 14-day screening period, eligible patients continued to receive their current tacrolimus formulation at an unchanged dose. Following reevaluation for inclusion/exclusion criteria, patients were then randomized to remain on their current tacrolimus preparation or to switch to the alternative formulation on a milligram for milligram basis. During period 1 (days 1–14), patients in sequence 1 received reference tacrolimus (Prograf®, Astellas Pharma Inc., Deerfield, IL, USA) and patients in sequence 2 received generic tacrolimus. During period 2 (days 15–28), the two groups crossed over to receive the alternative preparation. The generic formulation was Sandoz tacrolimus (Sandoz Inc., Princeton, NJ, USA). Patients were instructed to take their study medication twice daily at 12 h intervals, at the same time each day. No food was allowed until 2 h post-dose.

### Study objective

The primary objective of the study was to estimate the ratio of AUC_0–12h_ and C_max_ at steady state for generic versus reference tacrolimus in stable renal transplant patients using data from days 14 and 28 of the study.

### Patient population

Adult (≥18 years) recipients of a first or second kidney transplant at least 6 months prior to study entry who were receiving either generic tacrolimus (Sandoz) or reference tacrolimus were eligible for inclusion if they were receiving a stable dose of tacrolimus. Tacrolimus dose was to remain unchanged throughout the study. See Supplementary Methods for additional eligibility criteria.

### Pharmacokinetic and clinical assessments (see Supplementary Methods)

Pharmacokinetic testing was performed on days 7, 14, 21 and 28. Blood samples were collected pre-dose (C_0_) and at 0.5, 1, 1.5, 1.75, 2, 3, 4, 8 and 12 h after dosing. Samples were analyzed in a blinded manner at a central laboratory.

### Statistical analysis

The primary pharmacokinetic variables were dose-normalized AUC_0-t_ (i.e. AUC_0–12h_) and C_max_ at days 14 and 28. The sample size calculation estimated that 50 patients would be sufficient to meet the 80–125% confidence limits for the ratio of AUC_0–12h_ and C_max_ of generic tacrolimus to reference drug with a statistical power of at least 80%, assuming an intrasubject variation of 25% for AUC_0-t_ and C_max_, an intersubject variability of 50% and a ratio for the generic to the reference tacrolimus formulation of approximately 110%. Allowing for 10 drop-outs or nonevaluable patients, 60 patients were required. The assumptions of 25% for intrasubject variation and 110% for the ratio were derived from a previous pharmacokinetic comparison of the generic preparation versus the reference preparation in healthy volunteers. The assumed intersubject variability of 50% was based on a literature search of trials in transplant recipients.

Values for AUC_0–12h_ and C_max_ were dose-normalized by dividing the observed value by the dose recorded at the closest time to each of the measurements. These dose-normalized values were then log-transformed before statistical analyses. The ratios of geometric means (90% CI for the ratio) of AUC_0–12h_ and C_max_ at steady state of generic to reference tacrolimus were obtained by backtransforming the least squares (LS) mean difference (90% CI of the LS mean difference). The ratio of geometric mean and the associated 90% CI for the steady-state ratio of AUC_0–12h_ and C_max_ of generic to reference tacrolimus were calculated using data from days 14 and 28 based on an analysis of variance (ANOVA) model. The ANOVA model had fixed factors for treatment, period and sequence, and a random factor for subject effect (nested within sequences). To compare the bioavailability of generic to reference tacrolimus, the 90% CIs of the ratios of geometric means of AUC_0–12h_ and C_max_ were assessed relative to the interval (80%, 125%). The same analyses were performed to compare bioequivalence of each formulation with itself at different time points, based on data obtained on day 7 versus day 14, and on day 21 versus day 28.

To evaluate the intrapatient pharmacokinetic variability of each formulation, AUC_0-12h_, C_max_ and C_0_ were assessed at days 7, 14, 21 and 28. Intrapatient variability was assessed by a calculation of the coefficient of variation (CV, defined as standard deviation/mean × 100) and expressed as mean ± standard error of the mean (SD) (95% CI) for each formulation.

Pharmacokinetic variables were determined using noncompartmental methods. Unless otherwise specified, all statistical tests were conducted against a two-sided alternative hypothesis employing a significance level of 0.05. All statistical analyses were performed by iCS using SAS® Version 9.2.

## Results

### Patient population

**Figure 2:**
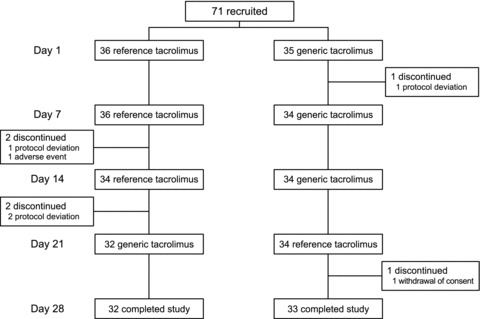
Patient disposition

Seventy-one patients were recruited to the study and comprised the safety and intent-to-treat (ITT) populations. Three patients were excluded from the pharmacokinetic analysis due to protocol deviations: one patient was dispensed only 45 tablets instead of the planned 70, and substituted his own medication when he ran out of study medication; one did not have assessments performed according to protocol; and one was not on a stable twice-daily dose of study drug prior to entry. The pharmacokinetic population thus comprised 68 patients. A total of 65 patients (91.5%) completed the study (Figure 2). Six patients discontinued the study (four in sequence 1 and two in sequence 2), with one patient discontinuing due to adverse events, two due to consent withdrawal and three for protocol deviations. Two of the discontinuing patients were also protocol violators; results from the other four discontinuing patients were included in individual analyses if the relevant data had been recorded prior to study withdrawal.

The mean (SD) age was 52.1 (12.5) years. More than half the patients (n = 43, 60.6%) were male and 60.6% (n = 43) were Caucasian. Thirty patients had diabetes (42.3%). The mean (SD) time posttransplant was 4.1 (3.3) years (median 3.5 years, range 0.6–15.3). Thirty patients had received a graft from a deceased donor (42.3%). Thirty patients (42.3%) were receiving corticosteroids and 70 were receiving mycophenolic acid (98.6%) at baseline and throughout the study.

### Efficacy and safety

No graft losses or episodes of rejection occurred during the study. Eight patients (11.9%) experienced a total of nine adverse events while receiving generic tacrolimus, compared to 12 patients (17.9%) who experienced 21 adverse events during administration of the reference preparation. The only adverse event to occur in more than one patient under generic tacrolimus was oropharyngeal pain (n = 2). During treatment with reference tacrolimus, vomiting (4), nausea (2), diarrhea (2) and headache (3) occurred in more than one patient. No unexpected adverse events were observed, and most adverse events were mild and transient. One patient experienced three serious adverse events (headache, mild rash and squamous cell carcinoma) during treatment with the reference drug. None was considered to be related to study drug. One patient discontinued due to headache, nausea and vomiting during administration of reference tacrolimus. Hyperkalemia and elevated aspartate aminotransferase were each reported in one patient during treatment with generic tacrolimus, while thrombocytopenia, hyperkalemia, hypokalemia and hyponatremia were reported in one case each during treatment with reference tacrolimus.

### Pharmacokinetics

**Figure 3:**
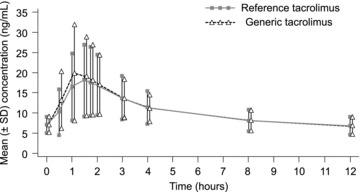
Time–concentration profiles for generic tacrolimus and reference tacrolimus Values are shown as mean (SD) for data obtained on days 14 and 28.

The mean (SD) tacrolimus dose at baseline was 5.7 (4.2) mg/day (median 4.0 mg/day, range 0.5–20.0 mg/day). All patients received an unchanged dose throughout the study. All measured tacrolimus trough concentrations were above the lower limit of quantitation (approximately 0.10 ng/mL). Figure 3 shows time–concentration profiles based on mean values of pharmacokinetic data obtained on days 14 and 28. There were no significant differences in AUC_0–12h_, C_0_, C_max_ or t_max_ between the generic and reference preparations based on mean values of data obtained on days 14 and 28 (Table 1). The ratios of geometric means (ANOVA) were 1.02 (90% CI of 97–108%, p = 0.486) for AUC_0–12h,_ 1.09 (90% CI 101–118%, p = 0.057) for C_max_ and 1.02 (90% CI of 95–109, p = 0.651) for C_12_.

**Table 1:** Dose-normalized AUC_0–12h_, dose-normalized C_max_, C_0_ and t_max_ for generic tacrolimus and reference tacrolimus

	Generic tacrolimus	Reference tacrolimus	p-Value^a^
Dose-normalized AUC_0–12h_ (ng*h/mL)	61.8 ± 40.6	60.0 ± 37.8	0.409
Dose-normalized C_max_ (ng/mL)	9.6 ± 5.5	9.1 ± 5.5	0.199
C_0_ (ng/mL)	7.3 ± 1.8	7.0 ± 2.1	0.354
T_max_ (hours)	1.5 ± 1.1	1.9 ± 1.3	0.073

Values are shown as mean ± SD of data obtained on days 14 and 28.

a ANOVA model with fixed factors for treatment, period and sequence and a random factor for subject effect (nested within sequences).

For generic tacrolimus and reference tacrolimus, the mean (SD) CV values were 13.4 (10.4)% versus 11.0 (9.8)% for AUC_0–12h_, 16.9 (15.5)% versus 17.9 (14.9)% for C_max_ and 13.2 (9.8)% and 11.1 (10.3)% for C_0_, respectively.

**Figure 4:**
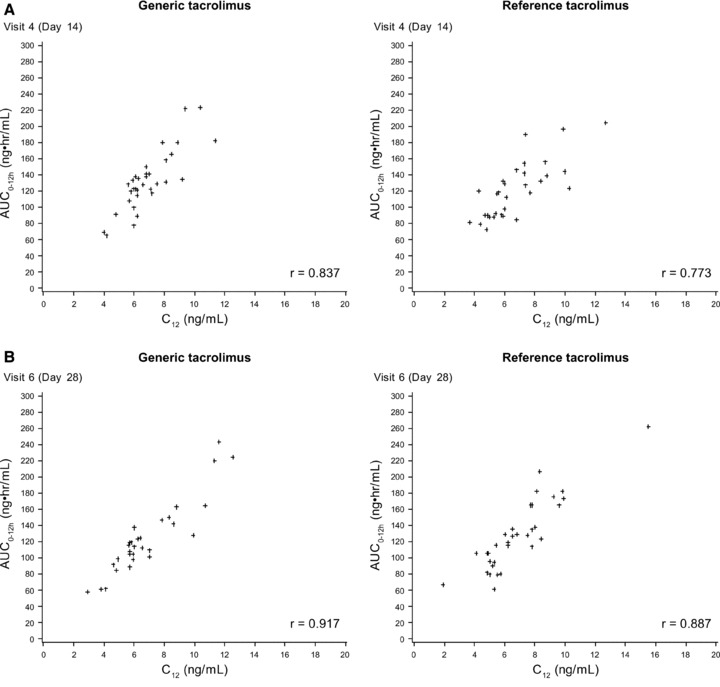
Scatter plots of AUC_0–12h_ versus trough concentration (C_12_) at (a) Day 14 (b) Day 28

Correlations (r values) between C_12_ and AUC_0–12h_ were 0.837 and 0.917 for generic tacrolimus at days 14 and 28, respectively, compared to 0.773 and 0.887 for reference tacrolimus (Figure 4).

### Intrapatient variability

The means of individual subject's CV values for AUC_0–12h_, C_max_ and C_0_ across all four pharmacokinetic assessments (days 7, 14, 21 and 28) were similar for generic or reference tacrolimus (Table 2). The ratios of geometric means for both generic tacrolimus and reference tacrolimus at day 7 versus day 14 and day 21 versus day 28 varied between 0.96 and 1.06 (Table 3).

**Table 2:** Intrapatient variability of dose-normalized pharmacokinetic parameters calculated as the mean of CV for individual subjects across all pharmacokinetic assessments (days 7, 14, 21 and 28), with CV defined as standard deviation/mean×100

	Generic tacrolimus	Reference tacrolimus
AUC_0–12h_, mean (SD), %	13.4 (10.4)	11.0 (9.8)
C_max_, mean (SD), %	16.9 (15.5)	17.9 (14.9)
C_0_, mean (SD), %	13.2 (9.8)	11.1 (10.3)

**Table 3:** Ratios of geometric means for AUC_0–12h_ and C_max_ for (a) generic tacrolimus and (b) reference tacrolimus at different time points

	Ratio of geometric means	90% CI	p-Value^a^
*(a) Generic tacrolimus*			
AUC_0–12h_			
Day 7 versus day 14	0.96	0.90, 1.03	0.327
Day 21 versus day 28	1.04	0.96, 1.12	0.450
C_max_			
Day 7 versus day 14	0.98	0.90, 1.07	0.735
Day 21 versus day 28	1.06	0.94, 1.19	0.423
*(b) Reference tacrolimus*			
AUC_0–12h_			
Day 7 versus day 14	0.96	0.91, 1.02	0.282
Day 21 versus day 28	0.98	0.91, 1.05	0.570
C_max_			
Day 7 versus day 14	0.96	0.88, 1.05	0.459
Day 21 versus day 28	1.00	0.89, 1.12	0.970

a Mixed model from the dose-normalized log-transformed values with measurement as a fixed factor and subject as the random effect.

## Discussion

To our knowledge, this is the first prospective study undertaken specifically to compare the pharmacokinetic characteristics of a generic tacrolimus preparation versus the reference drug in kidney transplant patients. The generic tacrolimus (Sandoz) showed a similar pharmacokinetic profile to reference tacrolimus, as assessed by a comparison of AUC_0–12h_, C_max_ and C_0_ concentration. These data indicate that the generic tacrolimus preparation is bioequivalent to reference tacrolimus in kidney transplant recipients according to the FDA guidelines. The findings will also apply to the branded generic tacrolimus Hecoria™, which has an identical formulation to the Sandoz generic.

The 90% CI values for the ratios of the geometric means of AUC_0–12h_ and C_max_ were each within the stipulated range of 80–125% (2), thus meeting the FDA criteria for bioequivalence (11). These criteria mean that the bioavailability of the generic product is not more than 20% higher or lower than the reference drug based on geometric means (13). The tighter criteria for AUC established by the EMA (90–111%) (12) and the Canadian Ministry of Health (90–112%) (14) for critical dose drugs such as tacrolimus were also met. The 90% CI values for the ratios for C_max_ (101–118%) were also within the ranges specified by the FDA (11) and Canadian guidelines (14) (both 80–125%) and although outside the narrower range stipulated by the EMA guidelines for narrow therapeutic index drugs (90–111%), the EMA does not apply C_max_ requirements to drugs such as tacrolimus where C_max_ is not pivotal for efficacy, safety or therapeutic drug monitoring (12). Correlations between AUC_0–12h_ and trough concentration were similar between the two preparations, and r^2^ values for generic tacrolimus at day 14 (0.701) and 28 (0.841) were comparable with published values for reference tacrolimus in kidney transplant patients (15–17). Application of bioequivalence testing to both the generic and the reference tacrolimus preparations, comparing the ratios of geometric means for AUC_0–12h_ and C_max_ at different time points one week apart, illustrated that both formulations showed some variability (Table 3). Intrapatient variation in bioavailability, shown to be a risk factor for interstitial fibrosis/tubular atrophy (IF/TA) and graft loss in patients receiving calcineurin inhibitors (18,19), was approximately 13% for both AUC_0–12h_ and C_0_ using generic tacrolimus (∼11% for reference tacrolimus). This compares favorably with reports in the literature, which have cited 14–44% using twice-daily reference tacrolimus (20–22).

Previously, results from a single-dose, two-way crossover study of the same generic formulation (Sandoz) in healthy volunteers showed that the 90% CI values for the ratios of geometric means of AUC_0-t_ and C_max_ were within the FDA criteria for bioequivalence in both the fed state (AUC_0-t_ 0.96 [90% CI 0.92, 1.00]; C_max_ 0.96 [0.90, 1.02]) and the fasting state (AUC_0-t_ 1.11 [90% CI 1.05, 1,22]; C_max_ 1.10 [1.02–1.19]) (23). Elsewhere, the mean difference in AUC values for four generic tacrolimus preparations approved by the FDA versus reference tacrolimus has been calculated to be 4% (range 0–11%) in fasting volunteers and 4.5% (range 2–6%) in the fed state (24). Steady-state dosing in transplant recipients, as in the current study, more accurately reflects the clinical situation, but in view of the known effect of food on tacrolimus pharmacokinetics (25), it is encouraging that bioequivalence criteria are met under both fed and fasting conditions following single-dose administration in volunteers. In our trial, the effect of food was controlled for by administering tacrolimus 2 h prior to eating.

The results of this pharmacokinetic analysis would suggest that clinical outcomes are likely to be similar using the generic tacrolimus (Sandoz) or the reference drug. In a prospective, observational study of conversion from reference to generic tacrolimus (Sandoz) in 70 patients from four centers, McDevitt-Potter et al. reported only minor changes in mean tacrolimus dose (4.4–4.5 mg, p = 0.89) and mean tacrolimus trough concentrations (5.8–5.9 ng/mL, p = 0.81) after conversion, although dose adjustments were more frequent than in the same patients at a point 6 months previously (21% of patients vs. 7% of patients) (26). This latter finding is not unexpected since more intensive monitoring would be likely following conversion to a generic preparation or other changes in the immunosuppression regimen. In two retrospective studies in which data were collected from kidney transplant patients converted from reference to generic tacrolimus (Sandoz), involving 45 patients (27) and 75 patients (28), respectively, the tacrolimus dose required to maintain therapeutic trough levels was similar with both preparations (27) and trough levels were maintained (27, 28). Across these trials and the present study, there was only one case of acute rejection following conversion, which occurred in a patient with a history of rejection (28). Limited data comparing *de novo* use of generic tacrolimus (Sandoz) or reference tacrolimus in kidney transplant patients showed no difference in dose requirements and trough concentration levels with both preparations (29). One case of biopsy-proven acute rejection has, however, been reported after an inadvertent conversion from reference to generic tacrolimus (Sandoz) (30). The rejection was not associated with a change in tacrolimus trough concentration and the authors suggested that the subsequent impairment in renal function was more likely to be due to underlying chronic allograft nephropathy (30), but the risk of inadvertent switch at the pharmacy level is of concern (9,10).

We are aware that this study addresses only conversion between the Sandoz formulation of generic tacrolimus and the reference drug although other formulations have now been developed. At the time the protocol was developed, the Sandoz preparation was the only commercially available generic tacrolimus. The current results cannot necessarily be extrapolated to other generic formulations. Also, all patients were required to take the study drug 2 h prior to food, and in a future trial of this type, it would be of interest to examine pharmacokinetic profiles with tacrolimus preparations taken at mealtimes. Although a washout period would standardly be used in a pharmacokinetic study of this type in healthy volunteers, it was not considered an acceptable risk in this population of kidney transplant patients since it would necessitate tacrolimus withdrawal. Use of a random sequence design and the 14-day treatment period would be expected to minimize any potential period effects.

While the required bioequivalence testing of generic tacrolimus formulations using a single dose in healthy volunteers provides useful data and meets regulatory requirements, tacrolimus metabolism differs in kidney transplant patients. Patients receive maintenance dosing and exhibit more rapid tacrolimus clearance than in healthy individuals, with a high rate of comorbid conditions, and receive other immunosuppressant agents that exert drug–drug interactions with tacrolimus. While there is no evidence that these factors vary between tacrolimus formulations, patient and clinical skepticism remains. The findings of the current study demonstrate that the Sandoz generic tacrolimus is bioequivalent to reference tacrolimus when assessed pharmacokinetically in a population of stable kidney transplant recipients.

## Abbreviations

ANOVA: analysis of variance
AUC: area under the curve
CI: confidence interval
CV: coefficient of variation
EMA: European Medicines Agency
FDA: Food and Drug Administration
IF/TA: interstitial fibrosis/tubular atrophy
ITT: intent-to-treat
LS: least squares
