# Supplementary material for: A Randomized Pharmacokinetic Study of Generic Tacrolimus Versus Reference Tacrolimus in Kidney Transplant Recipients
Source: Am J Transplant. 2012 Oct;12(10):2825–31. doi: 10.1111/j.1600-6143.2012.04174.x (PMC3472020; doi:10.1111/j.1600-6143.2012.04174.x)
Supplement: Supplementary file 1 [file ajt0012-2825-SD1.doc]

**A randomized pharmacokinetic study of generic tacrolimus versus reference tacrolimus in kidney transplant recipients**

RR Alloway, B Sadaka, J Trofe-Clark, A Wiland, RD Bloom

**Supplementary Methods**

*Study design and randomization*

Study drugs were supplied to all centers using the same lot numbers throughout the trial.

Each site was provided with a randomization sequence generated by a contract research organization (inVentiv Clinical Solutions, MD, USA) using an automated, validated system. Enrolled subjects were sequentially randomized according to this sequence. Drop-outs were replaced to preserve the sequence effect.

*Patient population*

A stable tacrolimus dose defined as one tacrolimus C_12_ level within the physician-defined target range within the past six months and one additional C_12_ level during the screening period within 30% of that range. Patients were required to have a body mass index ≥19 but ≤35kg/m^2^. Key exclusion criteria were evidence of any acute rejection; requirement for dialysis within the six months prior to study entry; receipt of a multiple organ transplant; presence of recurrent focal segmental glomerulosclerosis; severe gastrointestinal disturbance, diarrhea or diabetic gastroparesis; glomerular filtration rate (GFR) ≤35mL/min measured by the abbreviated Modification of Diet in Renal Disease formula (MDRD4); aspartate aminotransferase (AST), alanine aminotransferase (ALT), total bilirubin ≥3 times the upper limit of normal (ULN) or other evidence of severe liver disease; HIV positivity; and initiation of any medication that could interfere with tacrolimus blood levels and necessitate a change in tacrolimus dose during the study, including over-the-counter medications, herbal supplements, grapefruit or grapefruit juice.

*Pharmacokinetic and clinical assessments*

Prior to pharmacokinetic testing, patients were instructed to take their assigned tacrolimus preparation after an overnight fast. Full 12-hour fasting pharmacokinetic blood sampling took place approximately 12 hours after the evening dose. Blinded blood analysis was performed by Clinical Reference Laboratory Global Services, Lenexa, KS, USA. Tacrolimus blood concentrations were determined using a validated liquid chromatography tandem mass spectrometry assay with a lower limit of quantitation of approximately 0.10ng/mL.

At all post-randomization study visits, vital signs were recorded and data were collected on adverse events, concomitant medications or significant non-drug therapies, immunosuppressive medication, any changes or interruptions to the study medication, and the occurrence of graft rejection, graft biopsy or graft loss.

*Statistical analysis*

The pharmacokinetic analysis set included all patients to whom study medication had been assigned and who provided evaluable pharmacokinetic data. If a pharmacokinetic parameter could not be determined for one period, the corresponding patient was excluded from the analysis for that particular parameter. The safety population included all patients who received at least one dose of study medication.

*Study conduct*

The study was conducted in compliance with Good Clinical Practice and the ethical principles laid down in the Declaration of Helsinki after approval from the Institutional Review Board (IRB) for each center. Written informed consent was obtained from all patients.
